# Prevalence and factors associated with burnout syndrome in Peruvian health professionals before the COVID-19 pandemic: A systematic review

**DOI:** 10.1016/j.heliyon.2024.e30125

**Published:** 2024-04-27

**Authors:** Rosario M. Yslado Mendez, Junior Sanchez-Broncano, Gina D. Mendoza Ramirez, David Villarreal-Zegarra

**Affiliations:** a Universidad Nacional Santiago Antúnez de Mayolo, Ancash, Peru; b Escuela de Psicología, Universidad Continental, Lima, Peru

**Keywords:** Burnout, Health professional, Occupational health, Systematic review, Peru

## Abstract

**Introduction:**

Burnout syndrome (BS) is a prevalent occupational health problem in health professionals. To describe the prevalence and factors associated with BS in Peruvian health professionals.

**Method:**

A systematic review and meta-analysis were performed. The key terms “burnout” and “professional exhaustion” were used with words related to Peru. The databases consulted were LILACS/Virtual Health Library, Medline/PubMed, Science Direct, EBSCO, Scopus, SciELO, and RENATI-SUNEDU; articles published between January 2000 to December 2020 were considered for inclusion. Methodological quality was evaluated using the Newcastle–Ottawa scale.

**Results:**

Thirty studies were identified (8 scientific articles and 22 graduate theses). The median sample size was 78, with an interquartile range of 50–110. A meta-analysis was performed to calculate a dichotomic prevalence of burnout syndrome in health professionals of 25 % (95%CI: 9 %–45 %; I2 = 97.14 %; 5 studies). Also, our meta-analysis estimated the overall prevalence of mild burnout (27 %; 95%CI: 16%–41 %; I2 = 96.50 %), moderate burnout (48 %; 95%CI: 32%–65 %; I2 = 97.54 %), and severe burnout (17 %; 95%CI: 10%–24 %; I2 = 92.13 %; 18 studies). We present meta-analyses by region, profession, hospital area, and by dimension of the Maslach Burnout Inventory. Overall, the studies presented adequate levels of quality in 96.7 % of the included studies (n = 29). In addition, our narrative review of factors associated with BS and its three dimensions identified that different studies find associations with labor, socio-demographic, individual, and out-of-work factors.

**Conclusions:**

There is a higher prevalence of moderate BS in Peruvian health professionals at MINSA and EsSalud hospitals in Peru, with severity differing by region of Peru, type of profession, work area, and dimensions of BS.

## 1 Introduction

Burnout syndrome (BS), which comprises three dimensions, emotional fatigue, depersonalization, and personal fulfillment, is a serious public health problem that significantly affects health professionals exposed to work/care overload. BS begins with daily exposure to multiple occupational risks [1], inadequate working conditions [2], and interactions with patients (those who are terminally ill and critical and have a high probability of dying), their coworkers, and family members [3,4]. The negative consequences of BS manifest in the overall health of the affected professional. At the physical level, individuals with BS present migraines, headaches, muscle aches, gastrointestinal discomfort, hypertension, urticaria, asthma, tachycardia, and chronic fatigue, and psychological manifestations include frustration, irritability, anxiety, depression, low self-esteem, feelings of inefficiency, aggressive behaviors, lack of concentration and even suicide [5]. In addition, BS generates problems relating to work organization, such as deterioration in work quality, high turnover, decreased effectiveness and efficiency, conflicts between members of the organization, and recurrent absenteeism due to physical illnesses, which generates high costs for organizations [[6], [7], [8]].

For this research, we considered the studies that have used the MBI-HSS and the MBI to measure BS in health professionals, which are theoretically and epistemologically based on the psychosocial model, which conceptualizes BS as a multidimensional construct and a process that unfolds through the interaction between characteristics of the work environment and those of the individual. It is a three-dimensional model with three dimensions that assess cognitive aspects (occupational efficacy), emotional aspects (emotional exhaustion), and attitudinal aspects (depersonalization) [9]. In addition, social exchange theories suggest that individuals engage in social comparisons when establishing interpersonal relationships, which can lead to perceptions of inequity and unfairness between the resources invested and the pay and recognition received, lack of control over the outcomes of their work actions, prolonged exposure to excessive job demands, and others [10]. The organizational theory also explains the etiology of BS, claiming that issues stemming from organizational structure, climate, and culture, stressors in the organizational context, insufficient socio-labor support, coupled with coping strategies applied or not in the face of threatening work situations, are implicated in the origin and chronicity of burnout [10]. The reason for choosing this theory and not others is that it is one of the most widely used theories on burnout worldwide [4,[10], [11], [12]], and it has managed to divide the complex phenomenon of BS into three major components (emotional exhaustion, depersonalization, and personal accomplishment).

The prevalence of BS among healthcare professionals worldwide may vary depending on different specialties, countries, contexts, and other factors. A study in more than 30 countries found that the global prevalence of burnout among nurses was 30.0 % [11]. The prevalence of BS or burnout subcomponents among physicians found in 45 countries was 67.0 % [12]. Before the COVID-19 pandemic, 2.6 % of health professionals in Ecuador were found to have BS, during the pandemic it was 47.8 %. In Spain, it also increased from 33.4 % to 43.4 %. During the pandemic, high rates of SB were also reported in other countries, such as Bulgaria (15.2 %), Japan and Indonesia (31.4 % and 26.8 %, respectively), Australia (29.5 %) and Kenya (45.8 %) [13]. That is, the context of the COVID-19 pandemic has affected health systems and health professionals worldwide, similar to what happened in Peru [13]; therefore, it is important to focus on the Peruvian context.

At the international level, several systematic reviews have been conducted on BS in health professionals, most of which studied the prevalence, severity, causes, and associated factors in various populations of physicians and nurses from America, Europe, Asia, Africa, and Oceania. However, previous studies have not included Peruvian health professionals [[14], [15], [16], [17], [18], [19], [20], [21], [22], [23]]. In Peru, it is estimated that the prevalence of BS in physicians and nurses is 3.7 % and 2.1 %, respectively, based on a national survey conducted by the National Superintendence of Health [1], and there are no current data on the status of BS in professionals. In addition, the aforementioned survey measured only the prevalence of, not the factors associated with, BS in physicians and nurses. Therefore, there is no national information for other health professionals.

There has not yet been a study that groups the sociodemographic, individual, and intra- and extra-work factors associated with BS reported in studies, especially in Peruvian health professionals who work in hospitals of the Ministry of Health (MINSA for its acronym in Spanish) and the Social Security of Health (EsSalud for its acronym in Spanish). Global statistics and results at the national level can be used to not only identify many realities but also provide a broader picture of how this syndrome affects health professionals in Peru. This knowledge gap will be addressed with the results obtained in this research.

Based on the above, the aim study was to determine the factors associated with BS in Peruvian health professionals and determined the prevalence of BS based on regions, professions, hospital areas, and dimensions before the pandemic. Our systematic review was addressing the following research questions:

● What is the prevalence of BS by region, profession, hospital area, and dimensions of BS?
● What sociodemographic, individual, and intra- and extra-work factors are associated with an increased risk of BS in health professionals at hospitals of the Ministry of Health and Social Security of Health in Peru?

## 2 Methods

### 2.1 Study design

A systematic review and meta-analyses were performed following the criteria established by Preferred Reporting Items for Systematic Reviews and Meta-Analysis (PRISMA) [24] (Supplementary material 1). The research protocol was registered in PROSPERO with the code CRD42020168390 (https://www.crd.york.ac.uk/prospero/display_record.php?ID=CRD42020168390).

### 2.2 Eligibility criteria

To determine the eligibility of the research question for a systematic review, PEO nomenclature (Population, Exposure, and Results), as recommended by the Joanna Briggs Institute 2015 [25], was applied:

● Population: health professionals (physicians, nurses, obstetricians, dentists, pharmaceutical chemists, medical technologists, psychologists, nutritionists, and social workers); older than 18 years of age; who work in level I, II, and III hospitals of MINSA and EsSalud. In addition, we included studies using the Maslach Burnout Inventory.
● Exposure: factors that could influence the development of BS were evaluated: sociodemographic, individual (personal), work, and extra-work factors.
● Outcome: BS and its dimensions were measured using the Maslach Burnout Inventory (MBI, MBI-HSS), original or version adapted to Spanish. We use the MBI because it is one of the most widely used instruments to measure BS in health professionals.

We only include studies that we were able to access in full text, and that has been written in English, Spanish and Portuguese. The present research was restricted to only the search for studies published in the period from January 2000 to December 2020 for the following reasons. First, BS has acquired greater social relevance, resulting in great research interest in Europe and Latin America; publications in this regard also increased by considering BS a psychosocial risk related exclusively to work activity [26]. Second, an inclusion criterion was the use of the MBI-HSS and MBI to measure BS in health professionals [27]. Third, another inclusion criterion was the measurement of BS before the COVID-19 pandemic; the World Health Organization [28] classified COVID-19 as a pandemic in March 2020. On the same date, the Presidency of the Republic of Peru, through SD: N ° 008–2020-SA [29], announced a health emergency in the national health system, constituting a new context for conducting research studies in public and occupational health.

### 2.3 Information sources

Our study conducted a review of many databases of scientific articles such as LILACS/Virtual Health Library, Medline/PubMed, Science Direct, EBSCO, Scopus, and SciELO. In addition, we searched grey literature because, in Peru, many scientific papers fail to publish their results in scientific articles. The grey literature search was carried out by looking for undergraduate and postgraduate theses in the National Repository of Research Works of the National Superintendence of Higher Education (RENATI-SUNEDU).

### 2.4 Search strategy

In the search strategy, free terms or keywords were used, such as “Health professionals AND Peru AND burnout”, combined with MeSH and DeCS terms and Boolean operators. We present the search strategy for each study in Supplementary material 2.

### 2.5 Selection process

Two independent reviewers reviewed the articles and decide whether to include the study. In situations of disagreement, a third reviewer decides on inclusion. We conduct two types of reviews. First, a review of the title and abstract of all studies. Second, we assessed the full text of studies that passed the first review.

### 2.6 Data collection process and data items

We prepare a data extraction sheet with the information of interest from the included studies (Supplementary material 3). For each included study, the following information was extracted: author and year of publication, region of Peru, sample size, BS diagnostic scores, population, and BS severity (mild, moderate, and severe). In addition, the mean, median, and standard deviation of the total BS score and the scores for the three dimensions, or the interquartile range (IQR), were extracted.

The data extraction sheet was tested in an extraction pilot with ten randomized articles, and the data were extracted by two researchers independently. The interobserver agreement was evaluated, with a kappa value between 0.60 and 1 for the main variables to be collected. A minimum concordance value of 0.80 was considered acceptable [30].

### 2.7 Study risk of bias assessment

Methodological quality was assessed using the Newcastle-Ottawa Scale (NOS) adapted for cross-sectional studies. Three domains were assessed: study selection, comparability, and outcomes [31], which are the main sources of bias in this type of study. The risk of bias was assessed by two investigators independently (a third investigator was consulted in case of initial disagreement). The median NOS score was calculated from the assessment. Scores equal to or greater than 5 points indicated acceptable quality [32]. In addition, interobserver agreement was assessed using the kappa statistic, with values above 0.85 considered adequate [30].

We assessed publication bias by generating funnel plots for each outcome, only if the meta-analysis included 10 or more studies. Each funnel plot analysis considered the inverse standard error, the logarithm of the estimated prevalence of the outcome, and the ratio to the overall estimate of the outcome. We defined publication bias as an asymmetry in the number of studies within the 95 % confidence interval of the overall estimate [33].

### 2.8 Synthesis methods

#### 2.8.1 Meta-analysis

A quantitative analysis was performed for each study, taking into account the prevalence and its respective 95 % confidence interval for each of the 3 outcomes assessed (mild, moderate and severe BS) [34]. This information was synthesised by random effects meta-analysis due to the variability in the measurement of the outcome of interest. A meta-analysis was performed for each of the outcomes. In addition, the meta-analysis used a double arcsine transformation to calculate the estimated global prevalence of BS to stabilise the variance, as several confidence intervals approached 0 % [35]. Heterogeneity was assessed using the I^2^ statistic, and heterogeneity was considered significant or substantial with an I^2^ greater than or equal to 50 % [36]. In addition, for each outcome evaluated, analyses were performed by regional subgroups (Ancash, Arequipa, Cusco, Huancavelica, Junín, Lambayeque, Lima and Moquegua), by profession, hospital area, and dimension (emotional fatigue, depersonalization, and personal fulfillment). The statistical programme Stata v.16 MP was used, with the command ‘metaprop’ [34].

#### 2.8.2 Factors associated

To better understand the data presented in the studies, we carried out a qualitative assessment of all the evidence collected. We describe the factors statistically associated with BS and its dimensions, regardless of the statistical test used in each study. A quantitative analysis of each factor associated with an increased risk of BS was not performed because of the methodological heterogeneity in the use of the MBI (original or adapted version; different cut-off points to define the severity of BS and its dimensions; in some studies, the psychometric properties of the instrument are not specified; very different samples between studies, i.e. professionals from different specialities, different work settings, etc.), and different socio-occupational contexts) and in the statistics (weak statistical tests, such as hypothesis tests and no effect size for the associations), which make the quantitative analysis of the variables studied difficult. In particular, heterogeneity is a common finding reported in other systematic reviews [4,15,23].

## 3 Results

### 3.1 Study selection

**Fig. 1:**
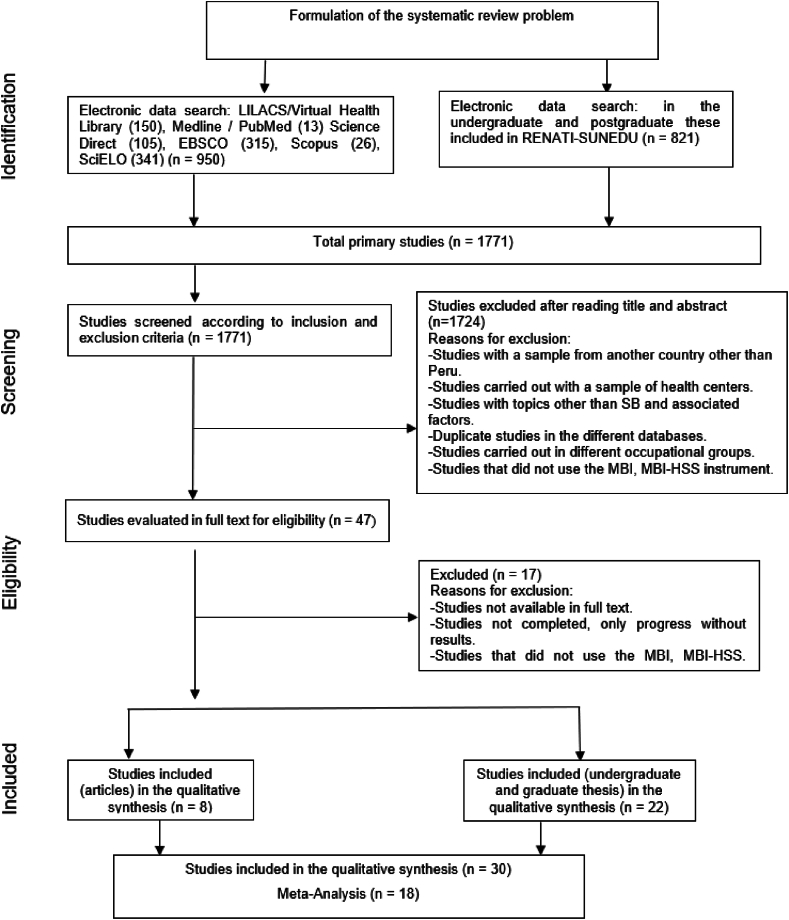
Flowchart.

We found a total of 1771 records after searching. After the title and abstract review only 47 records remained. The full-text review identified 8 scientific articles and 22 graduate theses. We included these 30 studies, however, we only included 18 studies in the meta-analysis. Fig. 1 shows the review process.

### 3.2 Study characteristics

42 % of the studies were conducted in Lima (n = 12), followed by Arequipa (4; 12.2 %), Ancash (3; 10 %), and La Libertad (3; 10 %). All studies were observational and cross-sectional in design. The median sample size was 78, with an interquartile range of 50–110. All studies used the Maslach Burnout Inventory but with different diagnostic criteria for the definition of burnout syndrome. Some studies did not even define the diagnosis of burnout syndrome, but only its domains. Also, 39.4 % of the studies included only nurses, 51.5 % included allied health professionals, and 9.1 % included only physicians. The median percentage of female participants was 74.5 % (RIC: 61.3 %–85.8 %), and the median percentage of male participants was 25.5 % (RIC: 14.3 %–38.8 %). The median of mean age was 39.2 years (ICR: 35–46.6 years), reported in only seven studies. The characteristics by each study is show in Table 1.

**Table 1:** Characteristics of each study included.

Author and year	Region	Sample size	BS diagnostic scores (Yes or No) or (Mild, moderate or severe)	Population	Health centers or hospitals	% of BS Mild	% of BS Moderate	% of BS Severe
Aedo Benites, 2015	La Libertad	78	NA	Hospital health personnel	La Esperanza Hospital I	NA	NA	NA
Albengrin Mendoza, 2008	Lima	60	Burnout no: 0–43; Trend: 44–87; BS presence: 88-plus	Ginecological – obstetrics – doctors	Guillermo Almenara Irigoyen National Hospital and Edgardo Rebagliati Martins National Hospital	11.1	75.6	13.3
Aldave Salazar, 2016	Lima	270	Diagnostic of BS: High scores in the dimensions of AE and DP, and low values in RP	Specialist doctors	Guillermo Almenara Irigoyen National Hospital	29.4	41.2	29.4
Ames Guerrero, 2014	Moquegua	64	Burnout low level: 0–24; Medium: 25–75; High: 76-plus	Hospital health personnel	Moquegua Regional Hospital	17	68.1	14.9
Arias-Gallegos, 2016	Arequipa	47	Burnout no: 0–43; Trend: 44–87; BS presence: 88-plus	Nurses	Hospitals of EsSalud and the Ministry of Health, Regional Institute of Neoplastic Diseases, Police Hospital, health centers in the metropolitan area of Arequipa and private health centers	3.8	90.6	5.6
Arias-Gallegos, 2017	Arequipa	213	Burnout low level: 0–44; Medium: 44–88; Severe: 89-plus	Hospital health personnel	Hospitals of EsSalud and the Ministry of Health, Regional Institute of Neoplastic Diseases, Police Hospital, health centers in the metropolitan area of Arequipa and private health centers	NA	NA	NA
Arteaga-Romani, 2014	Ica	238	Burnout no: 0–36; Trend: 37–72; BS presence: 73-plus	Hospital work personnel	Santa Maria del Socorro Hospital in Ica	78	2.4	19.6
Calcina Huayta, 2014	Arequipa	383	NA	Health personnel: doctors, nurses and obstetricians, who works in the Hospital	Honorio Delgado Espinoza Regional Hospital	57.9	14.5	72.6
Cardenas Talavera, 2013	Arequipa	75	Burnout low level (AE = or <18; DP = or <5; RP = or >40); Medium (SA 19–26; DP 6–9; RP 34–39); High (AE = or >27; PD 0 or >10; PR = <33).	Hospital health personnel	Belen Lambayeque Hospital	5	93	2
Carlos Cajo, 2018	Lambayeque	50	Low level burnout: 0–48; Medium: 49–61; High: 62-plus	Nurses	Víctor Larco District Regional Health Management Hospital	NA	NA	NA
Carranza Diaz, 2017	La Libertad	84	NA	Hospital work personnel	Dos de Mayo National Hospital	37.9	28.8	33.3
Chelin Pinco, 2017	Lima	80	Very low burnout level: 0–21; low: 22–42; so-so: 43–63; High: 64–84; Very High: 85-105	Nursing care of the hospitalization service	Daniel Alcides Carrion Hospital	NA	NA	NA
Chuco Tello, 2019	Junin	42	Burnout low level: 22–50; Medium: 51–80; High: 81-110	emergency care nurses	Applao Hospital	NA	NA	NA
Diaz Mercado, 2017	La Libertad	35	BS diagnostic ≥ 6 points: 1 point for each low score, 2 points for each intermediate score, and 3 points for each high score	Emergency healthcare personnel	Regional Teaching Hospital of Trujillo	3.5	95.3	1.2
Espinoza Rodriguez, 2011	Lima	82	NA	Assisting doctors	San Juan de Lurigancho Hospital	23.4	75.7	0.9
Estrada Mancisidor, 2016	Lima	98	Burnout low level: 21–48; Medium: 49–76; High: 77-105	Hospital health care personnel	National Hospital PNP Augusto B. Leguía	34.5	18.2	10
Flores Felipe, 2018	Lima	78	NA	Mental Health Nurses and Nursing Technicians	Edgardo Rebagliati Martins National Hospital	NA	NA	NA
Flores Huacaychuco, 2018	Lima	34	Burnout low level: 0–46; Medium: 47–67; High: 68-132	emergency care nurses	Daniel Alcides. National Hospital	NA	NA	NA
Gago, 2017	Huancayo	97	NA	nurses	General Hospital Daniel Alcides Carrion	86	14	0
Gomez Francia, 2015	Lambayeque	288	NA	healthcare personnel	Lambayeque Regional Hospital	42	29	29
Lopez Torres, 2017	Lime	111	Diagnostic of BS: High scores in the dimensions of AE [27–54] and DP [10–30], and low values in RP (0–33)	Health care nurses	Luis Negreiros Vega Hospital	80	10	10
Ore Flores, 2018	Huancavelica	30	NA	Outpatient nurse practitioners	Zacarias Correa Valdivia Regional Hospital	0	93.75	6.25
Pintado Chinchay, 2018	Piura	50	Burnout level low: 0–44; Medium: 45–89; High: 90-132	External office attending physicians	Hospital II Jorge Reátegui Piura	4.8	40.5	54.7
Robles Melgarejo, 2018	Lima	50	Diagnostic of BS: High scores in the dimensions of AE [27–54] and DP [10–30], and low values in RP [40–48]	Nurses	Lima Public Hospital	4.3	91.5	4.3
Silva Arquinego, 2018	Lima	47	Burnout low level: 0–53; Medium: 54–70; High: 71-plus	Nursing care in neonatal ICU	Cayetano Heredia National Hospital	NA	NA	NA
Solis-Chuquiyauri, 2016	Lima	43	Burnout low level: 0–48; Medium: 49–83; High: 84-132	Emergency care nurses	San Borja National Institute of Child Health	NA	NA	NA
Suasnabar Cayco, 2017	Lima	86	NA	nurses	National Institute of Neurological Diseases	NA	NA	NA
Yslado Mendez, 2011	Ancash	127	NA	Hospital appointed doctors, nurses and obstetrics.	Hospitals of Huari, Pomabamba and Sihuas of the Callejón de Conchuco	33.3	40	26.7
Yslado Mendez, 2013	Ancash	76	NA	Health personnel	Hospitals of Recuay, Huaraz, Carhuaz, Yungay and Caraz	NA	NA	NA
Yslado Mendez, 2019	Ancash	177	Burnout low level: 0–19; Medium: 20–32; High: 33-132	Health personnel	Chimbote Hospitals	12.2	78.6	9.2

AE: Emotional exhaustion; NA: Not available; PD: Depersonalization; IBM: Maslach Burnout Inventory; RP: Professional realization; BS: Burnout syndrome.

### 3.3 Risk of bias in studies

In general, studies presented adequate levels of quality in 96.7 % of the included studies (n = 29). Only one study presented inadequate quality levels [37], with scores below 5 points on the NOS scale. In general, the studies presented adequate levels of representative sample, exposure measurement and outcome measurement. The detailed description of the risk of bias assessment for each study can be found in Table 2.

**Table 2:** Methodological evaluation of each study with the Newcastle Ottawa tool for cross-sectional studies.

First author and year	Representative sample	Justified sample size	not surveyed	Exposure metering	comparability	Outcome measurement	statistical test	Total
Aedo Benites, 2015	★	★		★★		★★		6
Albengrin Mendoza, 2008	★			★★		★★		5
Aldave Salazar, 2016	★	★		★★		★★	★	7
Ames Guerrero, 2014	★			★★		★★		5
Arias-Gallegos, 2016	★			★★		★★		5
Arias-Gallegos, 2017	★			★★		★★	★	6
Arteaga-Romani, 2014	★			★★	★	★★	★	7
Calcina Huayta, 2014	★			★★		★★		5
Cardenas Talavera, 2013	★			★★		★★		5
Carlos Cajo, 2018	★			★★		★★		5
Carranza Diaz, 2017	★			★★		★★		5
Chelin Pinco, 2017	★			★★		★★		5
Chuco Tello, 2019	★			★★		★★		5
Diaz Mercado, 2017	★			★★		★★		5
Espinoza Rodriguez, 2011	★			★★		★★		5
Estrada Mancisidor, 2016	★		★	★★		★★		6
Flores Felipe, 2018	★			★★		★★	★	6
Flores Huacaychuco, 2018	★			★		★★		4
Gago, 2017	★		★	★★		★★		6
Gomez Francia, 2015	★	★		★★		★★		6
Lopez Torres, 2017	★			★★		★★		5
Ore Flores, 2018	★			★★		★★		5
Pintado Chinchay, 2018	★			★★		★★		5
Robles Melgarejo, 2018	★			★★		★★	★	6
Silva Arquinego, 2018	★			★★		★★		5
Solis-Chuquiyauri, 2016	★			★★		★★		5
Suasnabar Cayco, 2017	★			★★		★★		5
Yslado Mendez, 2011	★			★★		★★		5
Yslado Mendez, 2013	★	★		★★		★★		6
Yslado Mendez, 2019	★			★★		★	★	5

### 3.4 Meta-analysis of the prevalence of burnout syndrome

#### 3.4.1 Dicotomic prevalence

**Fig. 2:**
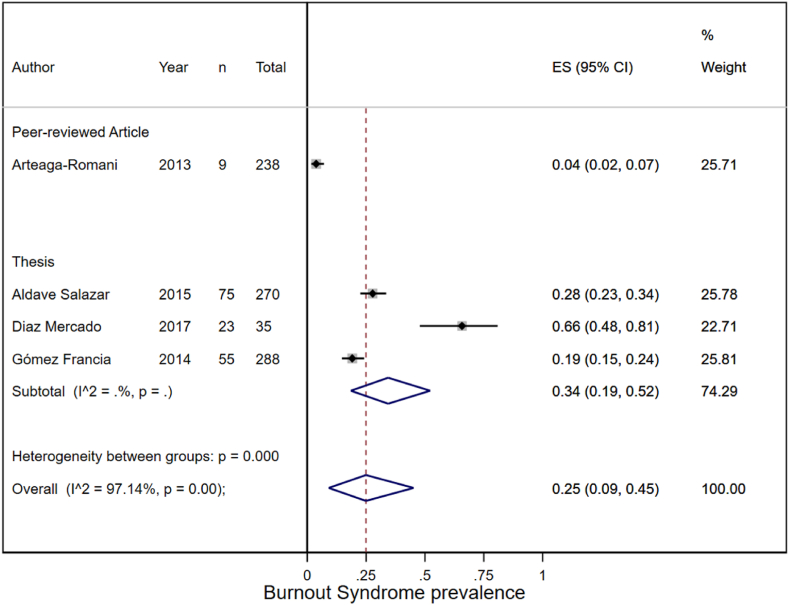
Meta-analysis of the prevalence of burnout syndrome.

Only four studies reported the prevalence of burnout (yes/no) in health professionals. A meta-analysis was performed to calculate an overall prevalence of burnout syndrome in health professionals of 25 % (95%CI: 9 %–45 %), with a heterogeneity of 97.14 % (see Fig. 2). In addition, a differentiated analysis was performed between peer-reviewed articles and dissertations, showing that the published article had a much lower prevalence than the three dissertations.

#### 3.4.2 Level prevalence

We found 18 studies that reported levels of burnout. Our meta-analysis estimated the overall prevalence of mild burnout (27 %; 95%CI: 16%–41 %; I^2^ = 96.50 %), moderate burnout (48 %; 95%CI: 32%–65 %; I^2^ = 97.54 %), and severe burnout (17 %; 95%CI: 10%–24 %; I^2^ = 92.13 %). We also made a separate estimate for scientific articles only. Table 3 shows the prevalence of each level of burnout (mild, moderate and severe) according to region, profession and hospital sector. We also performed a separate analysis for scientific articles only. Our analysis showed that the meta-analysis of peer-reviewed articles and dissertations overestimated the prevalence of burnout compared with the meta-analysis of peer-reviewed articles only. The dimensions of professional accomplishment and depersonalization were found to have a higher prevalence of severe levels compared to emotional exhaustion. The estimated prevalence for each burnout dimension is shown in Table 3. The funnel plots performed for meta-analyses with more than 10 studies identified publication bias in all cases.

**Table 3:** Meta-analysis according to the l f burnout syndrome, by region, profession and area.

		Peer-reviewed articles and thesis							Only peer-reviewed articles						
		Studies (n)	Mild		Moderate		Severe		Studies (n)	Mild		Moderate		Severe	
			ES (95 % CI)	I^2^	ES (95 % CI)	I^2^	ES (95 % CI)	I^2^		ES (95 % CI)	I^2^	ES (95 % CI)	I^2^	ES (95 % CI)	I^2^
Region	Ancash	3	0.60 (0.44–0.75)	89.20 %	0.11 (0.03–0.24)	90.16 %	0.27 (0.22–0.32)	12.71 %	3	0.60 (0.44–0.75)	89.20 %	0.11 (0.03–0.24)	90.16 %	0.27 (0.22–0.32)	12.71 %
	Arequipa	2	0.04 (0.02–0.06)	–	0.91 (0.87–0.94)	–	0.05 (0.03–0.08)	–	2	0.04 (0.02–0.06)	–	0.91 (0.87–0.94)	–	0.05 (0.03–0.08)	–
	Huancavelica	1	0.33 (0.17–0.53)	–	0.40 (0.23–0.59)	–	0.27 (0.12–0.46)	–	–	–	–	–	–	–	–
	Junin	1	0.05 (0.01–0.16)	–	0.40 (0.26–0.57)	–	0.55 (0.39–0.70)	–	–	–	–	–	–	–	–
	Lambayeque	1	0.16 (0.07–0.29)	–	0.68 (0.53–0.80)	–	0.16 (0.07–0.29)	–	1	0.16 (0.07–0.29)	–	0.68 (0.53–0.80)	–	0.16 (0.07–0.29)	–
	Lima	9	0.20 (0.07–0.38)	96.01 %	0.63 (0.43–0.81)	96.22 %	0.11 (0.02–0.24)	94.65 %	1	0.86 (0.72–0.95)	–	0.14 (0.05–0.28)	–	0.00 (0.00–0.08)	–
	Moquegua	1	0.00 (0.00–0.06)	–	0.94 (0.85–0.98)	–	0.06 (0.02–0.15)	–	–	–	–	–	–	–	–
Profession	Nurses	12	0.23 (0.09–0.41)	96.13 %	0.51 (0.31–0.72)	96.76 %	0.17 (0.07–0.30)	94.15 %	5	0.41 (0.08–0.87)	97.08 %	0.38 (0.07–0.77)	97.79 %	0.12 (0.02–0.27)	89.93 %
	Heath personnel	6	0.28 (0.06–0.56)	97.38 %	0.50 (0.17–0.83)	98.28 %	0.14 (0.06–0.24)	86.10 %	4	0.46 (0.08–0.87)	98.08 %	0.28 (0.00–0.83)	98.82 %	0.18 (0.05–0.37)	91.07 %
	Physicians	4	0.39 (0.12–0.71)	95.96 %	0.36 (0.04–0.79)	97.91 %	0.19 (0.06–0.38)	90.76 %	2	0.46 (0.24–0.69)	–	0.11 (0.05–0.18)	–	0.27 (0.18–0.36)	
Hospital area	General	13	0.26 (0.13–0.41)	96.80 %	0.50 (0.31–0.70)	97.99 %	0.15 (0.09–0.23)	92.31 %	6	0.42 (0.20–0.65)	97.13 %	0.31 (0.08–0.61)	98.31 %	0.19 (0.11–0.29)	86.50 %
	Outpatient	1	0.33 (0.17–0.53)	–	0.40 (0.23–0.59)	–	0.27 (0.12–0.46)	–	–	–	–	–	–	–	–
	Emergency	3	0.38 (0.00–0.89)	–	0.31 (0.14–0.51)	–	0.22 (0.00–0.66)	–	1	0.86 (0.72–0.95)	–	0.14 (0.05–0.28)	–	0.00 (0.00–0.08)	–
	ICU	1	0.17 (0.08–0.31)	–	0.68 (0.53–0.81)	–	0.15 (0.06–0.28)	–	–	–	–	–	–	–	–
Dimensions	Emotional exhaustion	18	0.29 (0.22–0.38)	92.50 %	0.29 (0.22–0.38)	92.07 %	0.16 (0.09–0.25)	95.07 %	4	0.63 (0.45–0.79)	89.06 %	0.22 (0.14–0.30)	60.28	0.14 (0.05–0.26)	84.01 %
	Despersonalize	18	0.39 (0.31–0.49)	92.79 %	0.31 (0.24–0.38)	88.78 %	0.25 (0.17–0.33)	92.59 %	4	0.55 (0.41–0.69)	82.40 %	0.55 (0.41–0.69)	82.40 %	0.25 (0.17–0.35)	64.14 %
	Professional accomplishment	18	0.30 (0.18–0.44)	97.00 %	0.32 (0.23–0.42)	94.47 %	0.29 (0.21–0.39)	93.63 %	4	0.65 (0.30–0.92)	97.09 %	0.12 (0.03–0.24)	86.19 %	0.21 (0.05–0.46)	94.64 %
Overall		18	0.27 (0.16–0.41)	96.50 %	0.48 (0.32–0.65)	97.54 %	0.17 (0.10–0.24)	92.13 %	7	0.46 (0.24–0.69)	97.32 %	0.29 (0.08–0.57)	98.20 %	0.17 (0.09–0.26)	88.45 %

Note: ES = effect size. I^2^= Heterogeneity.

### 3.5 Factors associated with BS

Table 4 shows that BS was more frequently associated with work factors such as a) regular or unfavorable work environment; inadequate structure, physical conditions, administrative policies and leadership styles (authoritarian, contradictory orders, pressure and labor demands); work stress and perceived work factors; insufficient socio-occupational (informative, instrumental and emotional) support; lack of control over work performed; and lack of recognition for job performance (n = 14); b) dysfunctional group behavior (inappropriate interpersonal relationships, low cordiality, cooperation and communication, injustice, and interpersonal and union conflicts) (n = 10); c) medium and low job satisfaction, perception of low remuneration, insufficient recognition of work performed (n = 6); d) excessive workload due to having more than 1 shift or job (n = 5); e) service time less than 5 years or more than 10 years (n = 5); and f) lower quality of professional life (n = 1). Among the sociodemographic factors, BS occurred indistinctly between women (n = 2) and men (n = 2). Regarding age, BS occurred in those younger than 40 years and older than 59 years (n = 2) and in widowed and separated doctors and nurses (n = 1). Furthermore, BS was associated with an individual or personal factor, such as job competence (n = 1) and pharmacological treatment (n = 1). The extra-work factors associated with BS were migrating for work (n = 1), family stress (n = 1), family burden (children) (n = 1), and lack of free time for recreational activities (n = 1).

**Table 4:** Factors associated with burnout syndrome and its domains, by individual study.

First author and year	Statistically significant factors associated with Burnout Syndrome	Statistically significant factors associated with emotional exhaustion	Statistically significant factors associated with depersonalization	Statistically significant factors associated with professional achievement
Aedo Benites, 2015	NE	State anxiety (direct and moderate, p < 0.001) and trait anxiety (direct and moderate, p < 0.001)	State anxiety (direct and moderate, p < 0.001) and trait anxiety (direct and moderate, p < 0.001)	State anxiety (inverse and moderate, p < 0.001) and trait anxiety (inverse and moderate, p < 0.001)
Albengrin Mendoza, 2008	NE	NE	NE	NE
Aldave Salazar, 2016	Sex (males are less likely to have BS OR:0.37 compared to women), have children (less likely to have BS OR:0.49 compared to not having children), work shift (having more than one shift is more likely of having BS OR:1.71 compared to having a work shift), work overload (OR:118.4), loss of control over what is done (OR:21.0), lack of recognition for the work performed (OR:8.0), loss of cordiality in the work environment (OR:10.2), injustice (OR:22.0)	NE	NE	NE
Ames Guerrero, 2014	Age (more high BS in ages 59 and over, p = 0.0023), sex (more high BS in women, p = 0.0038), marital status (more high BS in separated and widowed, p = 0.025), place of origin (more high BS in those from Moquegua and Tacna, p = 0.0621), length of service (more high BS in workers between 1 and 15 years of service), type of contract (more high BS in personal appointed, p = 0.0513), profession (more high BS in nurses, p = 0.016)	NE	NE	NE
Arias-Gallegos, 2016	Sex (men with higher BS score, p = 0.042), age (inverse, p=<0.001), length of service (direct, p = 0.012), profession (doctors and nurses with higher BS score, compared to psychology, nutrition, obstetrics and dentistry, p = 0.048)	Service time (direct, p = 0.022)	Sex (men with higher depersonalization score, p = 0.014), length of service (direct, p < 0.001), workplace (hospital with higher depersonalization compared to health center, p = 0.002), position (gerergic positions with less depersonalization, p = 0.003), profession (doctors and nurses greater depersonalization, compared to psychology, nutrition, obstetrics and dentistry, p = 0.001)	Service time (inverse, p = 0.025), position (hierarchical positions plus professional achievement, p = 0.004), profession (doctors and nurses less professional achievement, compared to psychology, nutrition, obstetrics and dentistry, p = 0.001)
Arias-Gallegos, 2017	Service time (Less than 5 years and greater than 10 years, higher BS score compared to 5–10 years, p = 0.034)	NES	NES	Time of service (Less than 5 years and greater than 10 years, higher professional achievement score compared to 5–10 years, p = 0.047)
Arteaga-Romani, 2014	Food intake (Higher score in BS in those with food intake, p = 0.045)	Food intake (Higher score in emotional exhaustion in those with food intake, p = 0.0333)	Chronic morbidity (Higher depersonalization score in those with chronic morbidity, p = 0.018), food intake (Higher depersonalization score in those with food intake, p = 0.007)	NES
Calcina Huayta, 2014	NE	Age (Greater emotional exhaustion among those aged 41–50, p = 0.0015), sex (more emotional exhaustion in women, p < 0.001), profession (midwives with higher emotional exhaustion, compared to doctors and nurses, p < 0.001), service time (those who work for more than 20 years plus emotional exhaustion, p < 0.001), employment status (hired more emotional exhaustion, p < 0.001), work area (those who work in the delivery room, ICU, and in the neonatal ICU greater emotional exhaustion, p < 0.001)	Age (greater depersonalization among those aged 41–50, p < 0.001), sex (more depersonalization in men, p < 0.001), marital status (single, greater depersonalization, p = 0.008), profession (doctors with greater depersonalization, in comparison with nurses and midwives, p < 0.001), length of service (those who work for more than 20 years, more depersonalization, p < 0.001), number of jobs (those who have more than 2 jobs, more depersonalization, p < 0.001), condition employment (hired greater depersonalization, p < 0.001), work area (those who work in more than 2 areas, ICU, and in neonatal ICU greater depersonalization, p < 0.001)	Age (less professional achievement among those aged 22–30, p < 0.001), sex (less professional achievement in men, p < 0.001), marital status (married, less professional achievement, p = 0.21), profession (midwives with lower professional achievement, compared to doctors and nurses, p < 0.001)
Cardenas Talavera, 2013	NE	NES	NES	NES
Carlos Cajo, 2018	Job satisfaction (moderate inverse, p = 0.01)	NE	NE	NE
Carranza Diaz, 2017	NE	NES	Self-actualization in work environment (inverse, p = 0.48), supervision in work environment (inverse, p = 0.048)	Labor involvement in the work environment (direct, p = 0.04), supervision in the work environment (direct, p = 0.04)
Chelin Pinco, 2017	Labor competition (strong direct, p < 0.001)	Labor competition (strong direct, p < 0.001)	Labor competition (strong direct, p < 0.001)	Labor competence (strong inverse, p < 0.001)
Chuco Tello, 2019	Work environment (strong inverse, p < 0.001), communication (moderate inverse, p < 0.001), leadership (inverse, p < 0.001), interpersonal relationships (inverse, p < 0.001), environmental conditions (inverse, p = 0.004)	NE	NE	NE
Diaz Mercado, 2017	Age (under 40 years of age, higher frequency of BS, p < 0.001), length of service (those who have been working <10 years with a higher frequency of BS, p < 0.001), mental burden (lower workload, lower frequency of BS, p = 0.02)	NE	NE	NE
Espinoza Rodriguez, 2011	Sex (men with a higher frequency of BS, p = 0.03), areas of stress (family and work stress, a higher frequency of BS compared to work stress, family stress or no stress, p = 0.005)	NE	NE	NE
Estrada Mancisidor, 2016	Job satisfaction (direct, p < 0.001)	Job satisfaction (direct, p < 0.001)	Job satisfaction (direct, p < 0.001)	Job satisfaction (direct, p < 0.001)
Flores Felipe, 2018	Workload (those who have a high workload are more likely to have BS, OR:2.5 compared to those who do not have a high workload), lack of workload (those who have a lack of workload more likely to have BS, OR:2.1), lack of encouragement and recognition (those with lack of encouragement and recognition more likely to have BS, OR:2.8), multiple jobs (those who have multiple jobs more likely to have BS, OR:1) .4), leadership style (those who participate in participative and authoritarian leadership have a higher frequency of BS, p < 0.001)	NE	NE	NE
Flores Huacaychuco, 2018	Job satisfaction (direct, p = 0.047)	NE	NE	NE
Gago, 2017	NE	NES	Work area (greater depersonalization in the ICU, compared to internal medicine, surgery, emergency, and traumatology, p = 0.018)	Work environment (direct, p < 0.001)
Gomez Francia, 2015	NE	NE	NE	NE
Lopez Torres, 2017	NE	Job performance (weak inverse, p = 0.037)	Job performance (weak inverse, p = 0.049)	NES
Ore Flores, 2018	Job satisfaction (inverse high, p < 0.001), physical conditions and/or comfort (inverse high, p < 0.001), labor and/or remunerative benefits (inverse high, p < 0.001), administrative policies (inverse high, p < 0.001), social relationships (moderate inverse, p < 0.001), personal development (high inverse, p < 0.001)	NE	NE	NE
Pintado Chinchay, 2018	NE	NE	NE	NE
Robles Melgarejo, 2018	NE	Age (higher score in emotional exhaustion in those who are 25–28 years old, compared to those who are 29–32 years old, p < 0.001), working conditions (inverse, p < 0.05), personal recognition and/or social (inverse, p < 0.05), economic benefits (inverse, p < 0.05)	NES	Working conditions (direct, p < 0.05)
Silva Arquinego, 2018	Stressing work factors (high direct, p < 0.001), factors related to pressure and demand (high direct, p < 0.001), organizational factors, human relations (high direct, p < 0.001), environmental factors (high direct, p < 0.001)	NE	NE	NE
Solís-Chuquiyauri, 2016^a^	Organizational climate (low inverse), organizational culture (inverse inverse), organizational design (inverse), human potential (inverse)	Organizational climate (inverse), organizational culture (inverse), organizational design (direct), human potential (direct)	Organizational climate (inverse), organizational culture (inverse), organizational design (inverse), human potential (inverse)	Organizational climate (direct), organizational culture (inverse), organizational design (inverse), human potential (inverse)
Suasnabar Cayco, 2017	Quality of life (inverse, p = 0.03)	NES	Workload (inverse, p = 0.002)	Intrinsic motivation (direct, p = 0.002)
Yslado Mendez, 2011	Housing tenure (Not having a home, higher frequency of final BS, p = 0.02), frequency of contradictory orders (always and sometimes higher frequency of final BS, p = 0.005), type of support (Not having informational, emotional, instrumental and others by co-workers more frequency of final BS, p = 0.04), activities in free hours outside the hospital (activities carried out in free hours and days outside the hospital, lower frequency of final BS, p = 0.003)	Individual factor (with individual factor plus emotional fatigue, p = 0.03), possibility of promotion (low possibility of promotion plus frequency of high emotional fatigue, p = 0.04), frequency of contradictory orders (always contradictory orders plus frequency of fatigue emotional exhaustion, p = 0.002), family conflicts (with family conflicts greater high emotional exhaustion, p = 0.02)	Home ownership (Not having a home has a higher depersonalization score, p = 0.03)	NES
Yslado Mendez, 2013	NE	NES	NES	Sex (women lower professional achievement score, p = 0.03)
Yslado Mendez, 2019^a^	Job satisfaction (inverse)	NE	NE	NE

a They do not report p value. NE: No statistical tests were performed to evaluate the association, NES: Non-statistically significant associations.

Regarding factors associated with the 3 dimensions of BS, emotional fatigue was frequently associated with work-related factors, such as a) regular work environment (n = 10), which includes work conditions (physical environment, insufficient remuneration and economic incentives, and insufficient personal/social recognition for work performed), unfavorable supervision, contradictory orders, few opportunities for promotion and professional development, insufficient socio-occupational (informative, instrumental and emotional) support, insufficient personal/social recognition; b) dysfunctional group behavior (interpersonal conflict) (n = 3); c) medium and low job satisfaction (n = 2); d) service time (less service time and longer than 20 years) (n = 2); e) excessive workload (n = 1); f) being hired (n = 1); lower professional performance (n = 1); and, h) work area (ICU, neonatal ICU, and delivery room) (n = 1). The associated sociodemographic factors were age (25–28 years and 41–50 years) (n = 2), sex (female) (n = 1), and type of profession (obstetrics) (n = 1). Among the associated individual or personal factors, state anxiety/trait anxiety (n = 1), chronic morbidity (n = 1), and perception of job competence (n = 2) were identified. The associated extra-work factor was family conflict (n = 1).

The depersonalization dimension was associated with the following work factors: a) a negative work environment (n = 5), which includes unfavorable supervision, medium-level self-realization, and insufficient socio-occupational (informational, instrumental, and emotional) support; b) excessive workload from working in 2 or more areas or working overtime (n = 5); c) time of service (less time of service and longer than 20 years) (n = 2); d) medium-level job satisfaction (n = 2); e) area of work (ICU) (n = 2); f) dysfunctional group behavior (interpersonal conflict and unions) (n = 2); g) being hired (n = 1); h) lower professional performance (n = 1); and i) having a high hierarchical position (n = 1). The associated sociodemographic factors were sex and male sex (n = 2), age 41–50 years (n = 1), marital status (single) (n = 1), and type of profession (doctors and nurses) (n = 2). The associated individual or personal factors were trait anxiety/state anxiety (n = 1), chronic morbidity (n = 1), and job competence (n = 1). The extra-work factors associated with depersonalization were family burden (spouse who does not work) (n = 1) and not having own home (n = 1).

The personal fulfillment dimension was more frequently associated with the following work factors: a) average work environment (n = 4), which includes unfavorable supervision, working conditions (physical, economic and/or psychosocial conditions), average work involvement, type of organizational design, type of organizational culture, and low-quality professional life; b) service time (less service time and longer than 10 years) (n = 2); c) dysfunctional group behavior (work conflicts, and inadequate interpersonal relationships) (n = 2); d) average job satisfaction (n = 1); e) being hired (n = 1); and f) having a high hierarchical position (n = 1). The associated sociodemographic factors were male sex (n = 2) and female sex (n = 1), age 22–30 years (n = 1), marital status (married) (n = 2), and type of profession (doctor, nurse or obstetrician) (n = 3). The associated individual factors were trait anxiety/state anxiety (n = 1), intrinsic motivation (n = 1), and job competence (n = 1). Additionally, the associated extra-work factor was quality of professional life (n = 1). A qualitative synthesis of the factors associated with BS and its 3 dimensions is provided in Table 5.

**Table 5:** Qualitative synthesis of associated factors related to Burnout syndrome and dimensions.

Burnout syndrome and dimensions	Labor factors	Sociodemographic factors	Individual factors	Out of work factors
Burnout syndrome	a) Background - Regular/poor work environment: • Organizational policy • Working conditions (physical, remuneration, incentives) • Authoritarian leadership style • Contradictory orders • Labor pressure and demand • Stress and perceived stressors • Insufficient socio-labor support • Lack of control over the work done • Lack of recognition for job performance - Dysfunctional group behavior • Inadequate interpersonal relationships • Interpersonal conflicts • Labor disputes - Medium/low job satisfaction - Excessive workload - Service time: <5 years,> 10 years - Be hired b) Consequences - Lower quality of professional life - Lower professional performance	- Sex: men and women - Profession: doctors and nurses - Marital status: widowed and separated	- Regular job competition - Pharmacological treatment (health)	- Not having your own home - Family stress - Family burden - Do not use free time in recreational activities
Emotional tiredness	a) Background - Regular work environment • Working conditions (physical, remuneration, incentives) • Adverse supervision • Contradictory orders • Insufficient socio-labor support • Little opportunity for promotion and professional development • Lack of personal/social recognition for job performance - Dysfunctional group behavior • Interpersonal conflicts -Medium and low job satisfaction - Excessive workload - Service time: <5 years,> 20 years -Be hired - Work area b) Consequences - Lower professional performance- Menor desempeño profesional	- Age: 25–28 years and 41–50 years - Sex:> women - Profession: obstetrician	- Regular job competition - State anxiety/trait anxiety (mental health) - Chronic morbidity (health) - Pharmacological treatment (health)	- Family problems
Depersonalization	a) Background - Regular work environment • Adverse supervision • Self-realization of media • Insufficient socio-labor support - Dysfunctional group behavior • Interpersonal conflicts • Labor disputes - Average job satisfaction - Excessive workload - Service time: <5 years,> 20 years -Having a hierarchical position - Be hired b) Consequences - Lower professional performance	- Age: 41–50 years - Sex:> male - Profession: doctors and nurses - Marital status: single	- Regular job competition - State anxiety/trait anxiety (mental health) - Chronic morbidity (health) - Pharmacological treatment (health)	- Family burden - Not having your own home
Low personal fulfillment	a) Background - Average work environment • Working conditions (physical, remuneration, incentives) • Adverse supervision • Medium labor involvement • Type of organizational design • Type of organizational culture - Dysfunctional group behavior • Inadequate interpersonal relationships • Labor disputes - Average job satisfaction - Excessive workload - Service time: <5 years,> 10 years - Have a hierarchical position - Be hired b) Consequences - Lower quality of professional life	-Age: 22–30 years - Sex:> male - Profession: doctors, nurses and obstetricians - Marital status Married	-Regular job competition -State anxiety/trait anxiety (health) -Intrinsic motivation	–

## 4 Discussion

### 4.1 Dichotomic prevalence

Our study found that in Peru, it is estimated that one in four health professionals has BS. We also found a higher prevalence of moderate and severe BS in the Peruvian healthcare group. This is a problem for health professionals and the Peruvian health system since the prevalence of BS increases accidents and decreases the quality of patient care [38]. Other systematic reviews conducted before the COVID-19 pandemic also reported a high prevalence of burnout among physicians [12,22], nurses [39], and other health professionals.

Our study found high heterogeneity of meta-analysis results by region, profession, hospital area, and dimension. The reasons for such high heterogeneity may be that they used modified versions of the MBI or versions of the MBI with inadequate measurement properties [40]. Also, the cut-off used by the different studies were very different, suggests that these elements should be considered sources of heterogeneity. Another source of heterogeneity is that our study included theses and original peer-reviewed articles, so it is possible that theses do not have the same quality standards as peer-reviewed articles. However, the quality analysis using the NOS suggests that the studies are generally of adequate methodological quality.

Our study found a prevalence of BS of 25 % in healthcare professionals, with results similar to the situation of BS in nurses, where it is estimated to be 30 % [11]. However, it is far from the prevalence in physicians, where BS is estimated to reach 67 % [12], which would affect the mental health of these professionals. Moreover, this problem has been exacerbated by the COVID-19 pandemic, during which the prevalence of BS among healthcare professionals increased significantly [8,[14], [15], [16], [17]].

### 4.2 Level prevalence of BS

Regarding the regional comparison, severe BS was more prevalent in cities at higher altitudes, such as Ancash, Huancavelica, and Junin, a finding that is partly explained by the precariousness of health services and the scarcity of health personnel to serve regions with high population density, causing overwork and permanent work stress [41,42]. Another study conducted in similar to another city located at a higher altitude such as La Paz in Bolivia found a similar prevalence (34 %) of BS among medical personnel [43].

In our study, the moderate BS was higher in nurses, while for physicians, the percentage with severe BS was higher than that for the other health professionals investigated. Also, similar results were reported in other studies conducted in Peru [[43], [44], [45]]. This result could be explained by moderating variables, such as characteristics of the medical profession, sociodemographic variables, and work stressors (high responsibility in hospitals of greater complexity, work overload, insufficient remuneration, low social support from colleagues or superiors). In addition, other studies from different parts of the world show that the majority of healthcare professionals have some form of BS [4,17,46].

Regarding the hospital area, our study identified that the professionals who work in ICU areas of hospitals have a higher prevalence of moderate BS, while personnel who work in outpatient and emergency departments have a higher prevalence of severe BS [47,48]. Other primary studies report that the prevalence of BS and its dimensions in professionals who work in emergency departments and ICU is high compared to that found in professionals who work in other areas [48,49]. Also, similar results are reported in other systematic reviews that include samples of emergency room nurses [4,50].

In addition, health personnel working in emergency departments, ICU and delivery rooms are exposed daily to unpredictable work demands, such as more hours of direct contact with patients, most of whom are in critical condition or prone to death, requiring greater physical and mental effort; in addition, these personnel are exposed to more aggression [51,52], which is a work stressor that conditions higher BS severity.

Our study found a severe and moderate levels of emotional exhaustion, depersonalization and low personal accomplishment. Another systematic review in nursing also found high levels of emotional exhaustion (28 %), and depersonalization (15 %) and low personal accomplishment (31 %) [53]. Also, another systematic review of healthcare workers during COVID-19 also found high levels of emotional exhaustion (51 %), depersonalization (52 %) and lack of personal accomplishment (28 %) [54]. The international evidence is similar to the Peruvian context. One possible explanation is that health systems in other countries have similar problems, such as limited buildings, excessive workloads, reduced numbers of health professionals and inadequate working conditions.

### 4.3 Factors associated with BS

This study supports Maslach's psychosocial and three-dimensional model of burnout and identifies work environment factors as major contributors to this condition [55,56]. Furthermore, our research emphasizes the etiology of burnout as a result of the interaction between the health professional and his or her work context (i.e., hospital ubication, type of profession, or hospital area), reinforcing the idea that burnout should be considered an occupational disease. This finding provides crucial evidence to promote legislation to protect the occupational and mental health of health professionals in Peru [57].

Regarding the proposed general objective, the results indicate that there are studies that support the association between the three dimensions of EB with the antecedent factors related to the work environment (regular, neutral or negative work environment; dysfunctional group behaviour, medium or low job satisfaction; and excessive workload), time of service less than 5 years or longer than 10 years and being hired), and consequent factors (lower job performance and quality of professional life). First, work factors posed the greater risk for the etiopathogenesis of BS in the Peruvian health community. Other systematic reviews found a relationship between work factors or working conditions with BS [[14], [15], [16], [17],[20], [21], [22],^46^] as well as between BS and the work environment (work performance, efficiency, and quality of service) [15,46,58,59]. Second, BS and its dimensions were associated with sociodemographic factors (sex, age, marital status, and type of profession), a finding that was also reported in previous studies conducted in Peru and other countries [4,14,15,43,46].

Third, personal or individual factors, such as perception of job competence (related to personal attributes and professional training) and having a health problem (anxiety and receiving pharmacological treatment) that involves morbidity, are associated with BS. Other studies, in addition to reporting the association between BS and work and sociodemographic factors, indicate that BS is associated with anxiety [4,60]. Less frequently, extra-work factors (family overload, family conflicts, not having own home, and no free time for recreational activities), as moderating variables, were associated with BS, emotional fatigue, and depersonalization. In this regard, in previous studies conducted in Peru and Mexico, family separation, number of children and relationship with a partner were associated with burnout [45,61]. The results of this study confirm the psychosocial model, which maintains that the origin and development of BS occurs mainly in the workplace [[62], [63], [64]], and theories of social and organizational exchange [26,65].

### 4.4 Limitations and strengths

The main strength of our study is that we estimated the prevalence of BS in Peruvian healthcare workers before the pandemic by region, hospital area, profession and dimensions. In addition, we consider our study to be the most comprehensive systematic review in the Peruvian context of BS among healthcare workers. Our study have a some limitations. First, many of the studies included were from the grey literature, which could bias the review as the quality of grey literature studies (dissertations) can be low. However, our review found that most of the studies were of adequate quality. Furthermore, the Cochrane recommendation is to use grey literature sources and include them in the systematic review [66]. Also, the inclusion of grey literature is justified by its significant contribution to knowledge for the systematic review, since it contains relevant information that, although currently found in university repositories, may eventually be published in peer-reviewed journals or books. This practice is recognized by several authors who emphasize the value of undergraduate and graduate theses as essential contributions to systematic reviews [67,68]. Second, our review did not include a meta-analysis of the associated factors because the included scientific articles and theses used heterogeneous methods and different analysis schemes. Third, our review only considered studies in which participants were assessed before the pandemic (March 15, 2020). Therefore, the context of the pandemic may have changed the working and personal conditions of healthcare workers, in which case our results could vary. Fourth, some of the sub-analyses included only a few studies, which means that any small change in prevalence may cause significant changes in the meta-analysis, which could bias our results. Researchers must consider our sub-analyses as exploratory aspects of the prevalence of burnout in Peru. Fifth, our results show high heterogeneity. Thus, the variability may be due to differences in the characteristics of the participants evaluated, the methodological design of the studies with small samples, and the working conditions of the participants. It is also possible that cross-cultural applications and adaptations of the MBI have been made in different studies [69]. Therefore, some studies may not be directly comparable. However, to reduce this heterogeneity and to make the studies more comparable, our systematic review only includes research using the MBI, which is the most widely used instrument for measuring BS worldwide [[69], [70], [71]]. Sixth, we only considered the use of the MBI, but other instruments allow the assessment of BS that assesses aspects other than the three traditional dimensions of the MBI. For example, the Copenhagen Burnout Inventory, which assesses personal, work-related, and client-related burnout [72]; the Oldenburg Burnout Inventory, which assesses exhaustion and detachment from work [73]; and other instruments that are less commonly used in the development of research on BS. However, a review found that the MBI is currently the most widely used instrument [74], and several studies have demonstrated its psychometric properties [40,75,76], so we believe that using only the MBI is justified. Finally, the studies found used different versions of the MBI and different cut-offs to determine levels and dimensions of burnout. Consequently, our findings may be inaccuracy in some cases due to the heterogeneity of the methodology used in these studies.

### 4.5 Public health implications

Our research shows the high prevalence of BS in health professionals before the pandemic. Therefore, our study could provide a baseline estimate for pre-pandemic assessments. There is evidence that the prevalence and intensity of emotional problems in the population increased during the pandemic [77]. In addition, our results contribute to knowing the prevalence of BS by the subgroups already mentioned, data that are necessary for the design of public policies for the protection of occupational health (license for temporary disability) and the promotion of well-being, prioritizing certain regions, occupational groups, work areas and dimensions of BS, to control and reduce BS and improve the lifestyles of health professionals and the quality of patient care. Importantly, a new socio-occupational context has been generated by the pandemic (COVID 19), which serves as a moderating variable that increases the frequency and intensity of BS (moderate to severe) in health professionals, as confirmed by various international studies [[78], [79], [80]].

Peru had a growing, stable, and sustainable economy, sufficient to face a health crisis and provide security to its population, during the pandemic Peru was one of the countries with the highest COVID-19 mortality rates in the world. However, during the pandemic, Peru was one of the countries with the highest COVID-19 mortality rates in the world, with rates concentrated in the regions with the highest poverty index [81]. In addition, Peru has a segmented and fragmented health system, with three ministries responsible for financing health care (the Ministry of Health, the Ministry of Labor, and the Ministry of Defense) and having the same steering capacity to establish health standards and policies, resulting in divided, unequally distributed, inadequate public health coverage, and limited geographic access [81]. It is therefore plausible that BS is also unequally distributed among different health professionals.

The prevalence of burnout also responds to structural factors in the health system. The Peruvian health system, characterized by its precariousness, faces adversities and significant organizational and structural weaknesses [82], such as deteriorating infrastructure, poor equipment, and uninsured temporary staff [83]. The over-concentration of doctors in the capital, which accounts for 50 % of the national total, exacerbates the shortage of qualified staff in more remote regions, increasing inequity in the distribution of resources, inequality, and the gap between supply and demand for health care [83]. These characteristics are similar in the health systems of other low- and middle-income countries [84].

### 4.6 Opportunities for future research

Our study only includes quantitative prevalence studies because they fit our general objective. However, we did not include qualitative studies and mixed methods, which could add to the understanding of the burnout phenomenon. We, therefore, call on researchers to conduct systematic reviews of qualitative studies or mixed methods on burnout in health professionals.

There is also a need for national assessments of burnout that consider sample sizes large enough to allow the generalization of results. On the one hand, there is also a need to focus on review research to assess the prevalence of burnout during the pandemic, as there is evidence of a higher prevalence of mental health problems and burnout. On the other hand, there is a need to develop longitudinal studies that assess the causal effect of burnout and related factors.

## 5 Conclusions

There is evidence to suggest the existence of a higher prevalence of moderate BS in Peruvian health professionals at MINSA and EsSalud hospitals in Peru, with severity differing by region of Peru, type of profession, hospital area, and dimensions of BS. However, there is a high heterogeneity in our findings, which should be taken into account when generalizing our results. In addition, our narrative review of factors associated with BS and its three dimensions identified that different studies find associations with labour, socio-demographic, individual, and out-of-work factors.

## Funding sources

Our study was funding by Universidad Nacional Santiago Antúnez de Mayolo (Rosario M. Yslado Mendez). The funder had no role in study design, data collection and analysis, decision to publish, or preparation of the manuscript.

## Ethics

Our study did not involve the collection of primary data on human subjects, but only a review of scientific articles and public lectures. The study, therefore, posed no ethical risk to participants.

## Availability of data

The database is attached in the following link: https://doi.org/10.6084/m9.figshare.25114118.v1.

## CRediT authorship contribution statement

**Rosario M. Yslado Mendez:** Writing – original draft, Methodology, Investigation, Funding acquisition, Conceptualization. **Junior D. Sanchez-Broncano:** Writing – review & editing, Validation, Methodology, Investigation, Data curation, Conceptualization. **Gina Mendoza Ramirez:** Writing – review & editing, Investigation, Data curation, Conceptualization. **David Villarreal-Zegarra:** Writing – original draft, Validation, Supervision, Software, Methodology, Investigation, Formal analysis, Data curation, Conceptualization.

## Declaration of generative AI and AI-assisted technologies in the writing process

We used DeepL to translate specific sections of the manuscript and Grammarly to improve the wording of certain sections. The final version of the manuscript was reviewed and approved by all authors.

## Declaration of competing interest

The authors declare the following financial interests/personal relationships which may be considered as potential competing interests:Rosario M. Yslado Mendez reports financial support was provided by E Universidad Nacional Santiago Antúnez de Mayolo.

## References

[1] Instituto Nacional de Estadística (2014). https://www.inei.gob.pe/media/MenuRecursivo/publicaciones_digitales/Est/Lib1192/.

[2] Canales-Vergara M., Valenzuela-Suazo S., Paravic-Klijn T. (2016 Jul 1). http://www.revista-enfermeria.unam.mx/ojs/index.php/enfermeriauniversitaria/article/view/82.

[3] Balcázar L.E., Montejo L.F., Ramírez Y.L. (2015 Oct 1). Prevalencia del síndrome de desgaste profesional en médicos residentes de un hospital de Mérida, Yucatán, México. Atención Fam..

[4] Albendín L., Gómez J.L., Cañadas-de la Fuente G.A., Cañadas G.R., San Luis C., Aguayo R. (2016 May 1). Prevalencia bayesiana y niveles de burnout en enfermería de urgencias. Una revisión sistemática. Rev. Latinoam. Psicol..

[5] Sánchez-Anguita A. (2008). Psicopatologías laborales. Universidad Pontificia de Salamanca.

[6] Matía Á.C., Cordero J., Mediavilla J.J., Pereda M.J., González M.L., González A. (2012 Sep 1). Evolución del burnout y variables asociadas en los médicos de atención primaria. Atención Primaria.

[7] Aguirre A.M., Quijano A.M. (2015). Síndrome por quemarse en el trabajo y variables familiares y laborales de los médicos generales de Bogotá. Una estrategia de calidad laboral. Elsevier Esp.

[8] Grau A., Flichtentrei D., Suñer R., Prats M., Braga F. (2009 Apr). Influencia de factores personales, profesionales y transnacionales en el síndrome de burnout en personal sanitario hispanoamericano y español (2007). Rev. Esp. Salud Publica.

[9] El síndrome de quemarse por el trabajo (burnout) | Ediciones Pirámide [Internet]. [cited 2023 Apr 26]. Available from: https://www.edicionespiramide.es/libro.php?id=1402776.

[10] Martínez A. (2010 Sep). El síndrome de Burnout. Evolución conceptual y estado actual de la cuestión. Vivat Acad Rev Comun.

[11] Meng‐Wei G., Fei‐Hong H., Yi‐Jie J., Wen T., Wan‐Qing Z., Hong‐Lin C. (2023 Sep). Global prevalence of nursing burnout syndrome and temporal trends for the last 10 years: a meta‐analysis of 94 studies covering over 30 countries. J. Clin. Nurs..

[12] Rotenstein L.S., Torre M., Ramos M.A., Rosales R.C., Guille C., Sen S. (2018 Sep 18). Prevalence of burnout among physicians: a systematic review. JAMA.

[13] Yslado RM, Nuñez L, Sánchez JD, De La Cruz-Valdiviano C, Soriano RM. Burnout Syndrome Among Health Professionals before and during the COVID-19 Pandemic..

[14] Ferreira da Rosa J., Bonow C.A., Cezar-Vaz M.R., Heck R.M., Xavier D.M. (2018 Jun 24). Burnout en profesionales de la salud hospitalaria. Rev Urug Enferm.

[15] Luengo C., Hidalgo N., Jara G., Rivera R. (2018). https://scholar.google.es/scholar?hl=es&as_sdt=0%2C5&q=burnout+en+profesionales+de+enfermeria+de+la+atencion+primaria+de+salud%3A+una+revision+sistematica+&btnG=#d=gs_qabs&u=%23p%3Ddx2eH9b-G80J.

[16] McCormack H.M., MacIntyre T.E., O'Shea D., Herring M.P., Campbell M.J. (2018). The prevalence and cause(s) of burnout among applied psychologists: a systematic review. Front Psychol [Internet].

[17] Dubale B.W., Friedman L.E., Chemali Z., Denninger J.W., Mehta D.H., Alem A. (2019 Sep 11). Systematic review of burnout among healthcare providers in sub-Saharan Africa. BMC Publ. Health.

[18] Zheng Q., Yang K., Wang X., Ou Z., Su X., Zhang J. (2019 Sep). Burnout among doctors in China through 2018: a protocol of systematic review and meta-analysis. Medicine (Baltim.).

[19] Bakusic J., Schaufeli W., Claes S., Godderis L. (2017 Jan). Stress, burnout and depression: a systematic review on DNA methylation mechanisms. J. Psychosom. Res..

[20] Vargas C., Cañadas G.A., Aguayo R., Fernández R., De La Fuente E.I. (2014 Jan). Which occupational risk factors are associated with burnout in nursing? A meta-analytic study. Int. J. Clin. Health Psychol..

[21] Pradas-Hernández L., Ariza T., Gómez-Urquiza J.L., Albendín-García L., De La Fuente E.I., Cañadas-De La Fuente G.A., Alameddine M. (2018 Apr 25). Prevalence of burnout in paediatric nurses: a systematic review and meta-analysis. PLoS One.

[22] Rodrigues H., Cobucci R., Oliveira A., Cabral J.V., Medeiros L., Gurgel K. (2018 Nov 12). Burnout syndrome among medical residents: a systematic review and meta-analysis. Junne FP. PLoS One.

[23] Rezaei S., Matin B.K., Hajizadeh M., Soroush A., Nouri B. (2018). Prevalence of burnout among nurses in Iran: a systematic review and meta-analysis. Int. Nurs. Rev..

[24] Moher D., Liberati A., Tetzlaff J., Altman D.G., The PRISMA Group (2009 Jul 21). Preferred reporting Items for systematic reviews and meta-analyses: the PRISMA Statement. PLoS Med..

[25] Peters M., Godfrey C., Mcinerney P., Soares C., Khalil H., Parker D. (2015).

[26] Gil-Monte P., Moreno-Jiménez B. (2007).

[27] Olivares-Faúndez V., Mena-Miranda L., Macía-Sepúlveda F., Jélvez-Wilke C. (2014). Validez factorial del Maslach Burnout Inventory Human Services (MBI-HSS) en profesionales chilenos. Univ Psychol..

[28] Organización Panamericana de la Salud (2020). La OMS caracteriza a COVID-19 como una pandemia - OPS/OMS | Organización Panamericana de la Salud. https://www.paho.org/es/noticias/11-3-2020-oms-caracteriza-covid-19-como-pandemia.

[29] Diario Oficial del Bicentenario El Peruano (2020). Decreto Supremo que declara en Emergencia Sanitaria a nivel nacional por el plazo de noventa (90) días calendario y dicta medidas de prevención y control del COVID-19-DECRETO SUPREMO-N° 008-2020-SA. http://busquedas.elperuano.pe/normaslegales/decreto-supremo-que-declara-en-emergencia-sanitaria-a-nivel-decreto-supremo-n-008-2020-sa-1863981-2/.

[30] Shea B.J., Reeves B.C., Wells G., Thuku M., Hamel C., Moran J. (2017 Sep 21). AMSTAR 2: a critical appraisal tool for systematic reviews that include randomised or non-randomised studies of healthcare interventions, or both. BMJ.

[31] Modesti P.A., Reboldi G., Cappuccio F.P., Agyemang C., Remuzzi G., Rapi S., Fuchs F.D. (2016 Jan 25).

[32] Jaung R., Robertson J., O'Grady G., Milne T., Rowbotham D., Bissett I.P. (2017 Jun). Limited evidence of abnormal intra-colonic pressure profiles in diverticular disease - a systematic review. Colorectal Dis..

[33] Centro Cochrane Iberoamérica (2011). Capítulo 10: análisis del sesgo de informe [Internet].

[34] Nyaga V.N., Arbyn M., Aerts M. (2014 Dec). Metaprop: a Stata command to perform meta-analysis of binomial data. Arch. Publ. Health.

[35] Barendregt J.J., Doi S.A., Lee Y.Y., Norman R.E., Vos T. (2013 Nov). Meta-analysis of prevalence. J. Epidemiol. Community Health.

[36] Centro Cochrane Iberoamérica (2011). Capítulo 9, análisis de los datos y realización de los metanálisis.

[37] Flores I. (2018). Síndrome de Burnout y su relación con Satisfacción laboral en Enfermeras del Servicio de Emergencia HNDAC, Callao 2018. http://repositorio.ucv.edu.pe/handle/UCV/18055.

[38] Dewa C.S., Jacobs P., Thanh N.X., Loong D. (2014 Jun 13). An estimate of the cost of burnout on early retirement and reduction in clinical hours of practicing physicians in Canada. BMC Health Serv. Res..

[39] Woo T., Ho R., Tang A., Tam W. (2020 Apr). Global prevalence of burnout symptoms among nurses: a systematic review and meta-analysis. J. Psychiatr. Res..

[40] Calderón-De La Cruz G.A., Merino-Soto C. (2020 Jun 23). Análisis de la estructura interna del Maslach Burnout Inventory (Human Service Survey) en médicos peruanos. Rev Cienc Salud..

[41] Chilca M., Pérez W., Loayza J., Huapaya F. (2018). nformación de recursos humanos del sector salud, Perú 2017.

[42] Instituto Nacional de Estadística e Informática. Censos Nacionales 2017: XII de Población, VII de Vivienda y III de Comunidades Indígenas. 2018 Resultados Definitivos de los Censos Nacionales 2017 – Censos Nacionales 2017. [cited 2023 Apr 26]. Available from: https://censo2017.inei.gob.pe/resultados-definitivos-de-los-censos-nacionales-2017/.

[43] Maticorena-Quevedo J., Beas R., Anduaga-Beramendi A., Mayta-Tristán P. (2016 May 24). Prevalencia del síndrome de burnout en médicos y enfermeras del Perú, Ensusalud 2014. Rev. Peru. Med. Exp. Salud Pública.

[44] Yslado R., Atoche R., Cermeño B., Rodriguez D., Sánchez J. (2013). Síndrome de Burnout y factores sociodemográficos-organizativos en profesionales de salud. Hospitales del callejón de Conchucos, Ancash, Perú - 2012. Rev Investig En Psicol.

[45] Solís-Cóndor R., Tantalean-del Águila M., Burgos-Aliaga R., Chambi-Torre J. (2017 Nov 30). Agotamiento profesional: prevalencia y factores asociados en médicos y enfermeras en siete regiones del Perú. An. Fac. Med..

[46] Chemali Z., Ezzeddine F.L., Gelaye B., Dossett M.L., Salameh J., Bizri M. (2019 Dec). Burnout among healthcare providers in the complex environment of the Middle East: a systematic review. BMC Publ. Health.

[47] Beltrán M.C., Mosquera J.V., Osorio J.S. (2020). https://repositorio.ibero.edu.co/entities/publication/8f37e095-b86e-45c5-8ad8-d31a9e4a062e.

[48] Madero-Zambrano K.P., Ayala-Jiménez D.P., Alvis-Estrada L., Bohórquez-Moreno C., Sanabria-Artunduaga M.E., Salas-Taborda H. (2022 Jan 28). Síndrome de agotamiento en profesionales de la salud posterior al primer año de pandemia por COVID-19. Ustasalud.

[49] Barquín F.I. (2019). Síndrome de Burnout en Urgencias. Rev Psicol SALUD [Internet].

[50] García-Iglesias J.J., Gómez-Salgado J., Fagundo-Rivera J., Romero-Martín M., Ortega-Moreno M., Navarro-Abal Y. (2022 Sep 23). Factores predictores de los niveles de burnout y work engagement en médicos y enfermeras: una revisión sistemática. Rev. Esp. Salud Publica.

[51] Aguilar L.A., Rivadeneyra N., del P. (2022). Burnout en las enfermeras de los hospitales públicos de la Región San Martín. https://repositorio.upeu.edu.pe/handle/20.500.12840/6083.

[52] Gamboa OM. Factores socioculturales y síndrome de burnout en el personal de enfermería de un centro de salud público, La Victoria - 2020 [Internet] [Tesis de licenciatura]. [Pimentel]: Universidad Señor de Sipán; 2022 [cited 2023 May 8]. Available from: http://repositorio.uss.edu.pe//handle/20.500.12802/9750.

[53] Monsalve-Reyes C.S., San Luis-Costas C., Gómez-Urquiza J.L., Albendín-García L., Aguayo R., Cañadas-De la Fuente G.A. (2018 May 10). Burnout syndrome and its prevalence in primary care nursing: a systematic review and meta-analysis. BMC Fam. Pract..

[54] Ghahramani S., Lankarani K.B., Yousefi M., Heydari K., Shahabi S., Azmand S. (2021). A systematic review and meta-analysis of burnout among healthcare workers during COVID-19. Front. Psychiatr..

[55] Maslach C., Jackson S.E., Leiter M.P. (1997). Evaluating Stress: A Book of Resources.

[56] Maslach C., Leiter M. (2021). Reversing Burnout. How to rekindle your passion for your work. Stanford Soc. Innovat. Rev..

[57] Instituto Nacional de Seguridad y Salud en el Trabajo. Portal INSST (2022). Síndrome de desgaste profesional (Burnout) como un problema relacionado con el trabajo. https://www.insst.es/el-instituto-al-dia/sindrome-de-desgaste-profesional-burnout.

[58] Carrillo-Esper R., Gómez k, Espinoza E. (2012). Síndrome de burnout en la práctica médica. Med. Int. Mex..

[59] Manging G. (2017). El síndrome de burnout: revisión literaria de su impacto en el bienestar y desempeño de los profesionales del sector salud. Ecuador]: Universidad de Especialidades Espíritu Santo.

[60] Gómez-Urquiza J., Monsalve-Reyes C., San Luis-Costas C., Fernández-Castillo R., Aguayo-Estremera R., Cañadas-de la Fuente G. (2017). Factores de riesgo y niveles de burnout en enfermeras de atención primaria: una revisión sistemática. Atención Primaria.

[61] Juárez-García A., Idrovo A., Camacho-Ávila A., Placencia Reyes O. (2014). Síndrome de burnout en población mexicana: Una revisión sistemática. Salud Ment.

[62] Gil-Monte P., Peiró J. (1999). Perspectivas teóricas y modelos interpretativos para el estudio del síndrome de quemarse por el trabajo. An Psicol..

[63] Gil-Monte P. (2006).

[64] Perrewé P.L., Ganster D.C., Perrewé Pamela L., Ganster Daniel C. (2004).

[65] Villarreal-Zegarra D., Lázaro-Illatopa W.I., Castillo-Blanco R., Cabieses B., Blukacz A., Bellido-Boza L. (2022 Oct 1). Relationship between job satisfaction, burnout syndrome and depressive symptoms in physicians: a cross-sectional study based on the employment demand–control model using structural equation modelling. BMJ Open.

[66] Higgins J., Thomas J., Chandler J., Cumpston M., Li T., Page M. (2022). Cochrane. Cochrane Handbook for Systematic Reviews of Interventions.

[67] Sánchez M., Navarro F., Sánchez-Meca J. (2022). Las Revisiones Sistemáticas y la Educación Basada en Evidencias. Espiral Cuad Profr.

[68] Villasis-Keever M.Á., Rendón-Macías M.E., García H., Miranda-Novales M.G., Escamilla-Núñez A. (2020 Jan 17). La revisión sistemática y el metaanálisis como herramientas de apoyo para la clínica y la investigación. Rev. Alerg. Mex..

[69] Soares J.P., Lopes R.H., Mendonça PB. de S., Silva C.R.D.V., Rodrigues C.C.F.M., Castro JL de (2022 Nov 1). Use of the Maslach burnout inventory among public health care professionals: protocol for a scoping review. JMIR Res Protoc.

[70] Forné C., Yuguero O. (2022 Aug 12). Factor structure of the Maslach burnout inventory human services survey in Spanish urgency healthcare personnel: a cross-sectional study. BMC Med. Educ..

[71] Meira-Silva V.S.T., ACTN Freire, Zinezzi D.P., Ribeiro F.C.R., Coutinho G.D., Lima I.M.B. (2022). Burnout syndrome in healthcare workers during the COVID-19 pandemic: a systematic review. Rev Bras Med Trab Publicacao Of Assoc Nac Med Trab-ANAMT..

[72] Moser C.M., Tietbohl-Santos B, Arenas DL, Xavier A, Ornell F., Borges R.B. (2023). Psychometric properties of the Brazilian Portuguese version of the Copenhagen Burnout Inventory (CBI) in healthcare professionals. Trends Psychiatry Psychother.

[73] Runyon C.R., Paniagua M.A., Dyrbye L.N. (2023 Jan 1). Exploring the validity based on internal structure of the Oldenburg burnout inventory – medical student (OLBI-MS). Teach. Learn. Med..

[74] Márquez-Lugo I., Mosquera-Quiñónez M., Ochoa-Granados C., Pacavita-Sánchez D., Palencia-Sánchez F., Riaño-Casallas M. (2021). https://www.ssrn.com/abstract=3841093.

[75] Calderón-de La Cruz G.A., Merino-Soto C., Juárez-García A., Dominguez-Lara S., Fernández-Arata M. (2020 Sep). ¿Es replicable la estructura factorial del Maslach Burnout Inventory Human Service Survey (MBI-HSS) en la profesión de enfermera del Perú?: un estudio nacional. Enferm Clínica.

[76] Yslado R.M., Sánchez-Broncano J., De La Cruz-Valdiviano C., Quiñones-Anaya I., Reynosa E. (2023 Oct 2). Psychometric properties of the Maslach burnout inventory in healthcare professionals. Ancash Region, Peru. F1000Research.

[77] Villarreal-Zegarra D., Reátegui-Rivera C.M., Otazú-Alfaro S., Yantas-Alcantara G., Soto-Becerra P., Melendez-Torres G.J. (2023 Mar 8). Estimated impact of the COVID-19 pandemic on the prevalence and treatment of depressive symptoms in Peru: an interrupted time series analysis in 2014–2021. Soc. Psychiatr. Psychiatr. Epidemiol..

[78] Azoulay E., De Waele J., Ferrer R., Staudinger T., Borkowska M., Povoa P. (2020 Dec). Symptoms of burnout in intensive care unit specialists facing the COVID-19 outbreak. Ann. Intensive Care.

[79] Barello S., Palamenghi L., Graffigna G. (2020 Aug). Burnout and somatic symptoms among frontline healthcare professionals at the peak of the Italian COVID-19 pandemic. Psychiatr. Res..

[80] Matsuo T., Kobayashi D., Taki F., Sakamoto F., Uehara Y., Mori N. (2020 Aug 4). Prevalence of health care worker burnout during the coronavirus disease 2019 (COVID-19) pandemic in Japan. JAMA Netw. Open.

[81] Gianella C., Gideon J., Romero M.J. (2021 Apr 3). What does COVID-19 tell us about the Peruvian health system?. Can J Dev Stud Rev Can Détudes Dév..

[82] Neyra-León J., Huancahuari-Nuñez J., Díaz-Monge J.C., Pinto J.A. (2021). The impact of COVID-19 in the healthcare workforce in Peru. J. Publ. Health Pol..

[83] Maguiña C. (2020 Mar 31). Reflexiones sobre el COVID-19, el Colegio Médico del Perú y la Salud Pública. Acta Méd. Peru..

[84] Ahmed S., Chase L.E., Wagnild J., Akhter N., Sturridge S., Clarke A. (2022 Apr 11). Community health workers and health equity in low- and middle-income countries: systematic review and recommendations for policy and practice. Int. J. Equity Health.

