# Supplementary material for: Prevalence and factors associated with burnout syndrome in Peruvian health professionals before the COVID-19 pandemic: A systematic review
Source: Heliyon. 2024 Apr 27;10(9):e30125. doi: 10.1016/j.heliyon.2024.e30125 (PMC11078627; doi:10.1016/j.heliyon.2024.e30125)
Supplement: Multimedia component 2 [file mmc2.docx]

**Supplementary material 2.** Database search strategy.

**Science Direct**

| #1 | (burnout OR "emotional exhaustion" OR "professional exhaustion" OR "psychological exhaustion" ) AND Peru |
| --- | --- |

**LILACS/ Biblioteca Virtual en Salud**

| #1 | (tw:(Burnout) OR tw:(“emotional exhaustion”) OR tw:(“professional exhaustion”) OR tw:(“psychological exhaustion”) OR tw:("agotamiento emocional") OR tw:("agotamiento profesional") OR tw:("agotamiento psicológico") OR tw:(“sindrome del quemado”)) |
| --- | --- |
| #2 | (tw:(“health Personnel”) OR tw:(“health Professional”) OR tw:(“Health Care Providers”) OR tw:(“Health Care Provider”) OR tw:(“Healthcare Providers”) OR tw:(“Healthcare Provider”) OR tw:(“Healthcare Workers”) OR tw:(“Healthcare Worker”) OR tw:(nurs*) OR tw:(Physician*) OR tw:(Dentist*) OR tw:(Nutritionist*) OR tw:(Obstetrician*) OR tw:(“Pharmaceutical chemist”) OR tw:(“Pharmaceutical chemists”) OR tw:(Psychologist*) OR tw:(“Social Assistant”) OR tw:(“Social Assistants”) OR tw:(“Medical technologist”) OR tw:(“Medical technologists”) OR tw:(“Personal de salud”) OR tw:(“Profesional de la salud”) OR tw:(“Proveedores de atención médica”) OR tw:(“Proveedor de atención médica”) OR tw:(“Trabajadores de la salud”) OR tw:(“Trabajador de la salud”) OR tw: (enfermer*) OR tw: (Médico*) OR tw: (Dentista*) OR tw:(Nutricionist*) OR tw:(Obstet*) OR tw:(“Químico farmacéutico”) OR tw: (“Químicos farmacéuticos”) OR tw: (Psicólog*) OR tw:(“Asistente social”) OR tw:(“Asistentes sociales”) OR tw:("Tecnólogo médico") OR tw:("Tecnólogos médicos") ) |
| #3 | (tw:(Peru) OR tw:(Perú) OR tw:(Amazonas) OR tw:(Ancash) OR tw:(Apurímac) OR tw:(Arequipa) OR tw:(Ayacucho) OR tw:(Cajamarca) OR tw:(Cusco) OR tw:(Callao) OR tw:(Huancavelica) OR tw:(Huánuco) OR tw:(Ica) OR tw:(Junín) OR tw:(“La Libertad) OR tw:(Lambayeque) OR tw:(Lima) OR tw:(Loreto) OR tw:(“Madre de Dios”) OR tw:(Moquegua) OR tw:(Pasco) OR tw:(Piura) OR tw:(Puno) OR tw:(“San Martín”) OR tw:(Tacna) OR tw:(Tumbes) OR tw:(Ucayali) ) |
| #4 | #1 AND #2 AND #3 |

**PUBMED/MEDLINE**

| #1 | “Burnout, Psychological”[mesh] OR “Burnout, Professional”[mesh] OR Burnout[tiab] OR “emotional exhaustion”[tiab] OR “professional exhaustion”[tiab] OR “psychological exhaustion”[tiab] |
| --- | --- |
| #2 | “Health Personnel”[mesh] OR “Health Personnel”[tiab] OR "Health Professional"[tiab] OR "Health Care Providers"[tiab] OR "Health Care Provider"[tiab] OR "Healthcare Providers"[tiab] OR "Healthcare Provider"[tiab] OR "Healthcare Workers"[tiab] OR "Healthcare Worker"[tiab] OR Nurs*[tiab] OR Physician*[tiab] OR Dentist*[tiab] OR Nutritionist*[tiab] OR Obstetrician*[tiab] OR “Pharmaceutical chemist”[tiab] OR “Pharmaceutical chemists”[tiab] OR Psychologist*[tiab] OR “Social Assistant”[tiab] OR “Social Assistants”[tiab] OR “Medical technologist”[tiab] OR “Medical technologists”[tiab] |
| #3 | Peru*[tiab] OR Peru[pl] OR “Peru”[mesh] OR Amazonas[tiab] OR Ancash[tiab] OR Apurímac[tiab] OR Arequipa[tiab] OR Ayacucho[tiab] OR Cajamarca[tiab] OR Cusco[tiab] OR Callao[tiab] OR Huancavelica[tiab] OR Huánuco[tiab] OR Ica[tiab] OR Junín[tiab] OR “La Libertad”[tiab] OR Lambayeque[tiab] OR Lima[tiab] OR Loreto[tiab] OR “Madre de Dios”[tiab] OR Moquegua[tiab] OR Pasco[tiab] OR Piura[tiab] OR Puno[tiab] OR “San Martín”[tiab] OR Tacna[tiab] OR Tumbes[tiab] OR Ucayali[tiab] |
| #4 | #1 AND #2 AND #3 |

**SCOPUS**

| #1 | TITLE-ABS-KEY ( burnout OR "emotional exhaustion" OR "professional exhaustion" OR "psychological exhaustion" ) |
| --- | --- |
| #2 | TITLE-ABS-KEY ( "Health Personnel" OR "Health Professional" OR "Health Care Providers" OR "Health Care Provider" OR "Healthcare Providers" OR "Healthcare Provider" OR "Healthcare Workers" OR "Healthcare Worker" OR nurs* OR physician* OR dentist* OR nutritionist* OR obstetrician* OR "Pharmaceutical chemist" OR "Pharmaceutical chemists" OR psychologist* OR "Social Assistant" OR "Social Assistants" OR "medical technologist" OR "medical technologists" ) |
| #3 | TITLE-ABS-KEY(Peru* OR Amazonas OR Ancash OR Apurímac OR Arequipa OR Ayacucho OR Cajamarca OR Cusco OR Callao OR Huancavelica OR Huánuco OR Ica OR Junín OR "La Libertad" OR Lambayeque OR Lima OR Loreto OR "Madre de Dios" OR Moquegua OR Pasco OR Piura OR Puno OR "San Martín" OR Tacna OR Tumbes OR Ucayali) |
| #4 | #1 AND #2 AND #3 |

**EBSCO**

| S1 | TI ( burnout OR "emotional exhaustion" OR "professional exhaustion" OR "psychological exhaustion" ) OR AB ( burnout OR "emotional exhaustion" OR "professional exhaustion" OR "psychological exhaustion" ) OR SU ( burnout OR "emotional exhaustion" OR "professional exhaustion" OR "psychological exhaustion" ) |
| --- | --- |
| S2 | TI ( "Health Personnel" OR "Health Professional" OR "Health Care Providers" OR "Health Care Provider" OR "Healthcare Providers" OR "Healthcare Provider" OR "Healthcare Workers" OR "Healthcare Worker" OR nurs* OR physician* OR dentist* OR nutritionist* OR obstetrician* OR "Pharmaceutical chemist" OR "Pharmaceutical chemists" OR psychologist* OR "Social Assistant" OR "Social Assistants" OR "medical technologist" OR "medical technologists" ) OR **A**B ( "Health Personnel" OR "Health Professional" OR "Health Care Providers" OR "Health Care Provider" OR "Healthcare Providers" OR "Healthcare Provider" OR "Healthcare Workers" OR "Healthcare Worker" OR nurs* OR physician* OR dentist* OR nutritionist* OR obstetrician* OR "Pharmaceutical chemist" OR "Pharmaceutical chemists" OR psychologist* OR "Social Assistant" OR "Social Assistants" OR "medical technologist" OR "medical technologists" ) OR SU ( "Health Personnel" OR "Health Professional" OR "Health Care Providers" OR "Health Care Provider" OR "Healthcare Providers" OR "Healthcare Provider" OR "Healthcare Workers" OR "Healthcare Worker" OR nurs* OR physician* OR dentist* OR nutritionist* OR obstetrician* OR "Pharmaceutical chemist" OR "Pharmaceutical chemists" OR psychologist* OR "Social Assistant" OR "Social Assistants" OR "medical technologist" OR "medical technologists") |
| S3 | TI ( Peru* OR Amazonas OR Ancash OR Apurímac OR Arequipa OR Ayacucho OR Cajamarca OR Cusco OR Callao OR Huancavelica OR Huánuco OR Ica OR Junín OR "La Libertad" OR Lambayeque OR Lima OR Loreto OR "Madre de Dios" OR Moquegua OR Pasco OR Piura OR Puno OR "San Martín" OR Tacna OR Tumbes OR Ucayali ) OR AB ( Peru* OR Amazonas OR Ancash OR Apurímac OR Arequipa OR Ayacucho OR Cajamarca OR Cusco OR Callao OR Huancavelica OR Huánuco OR Ica OR Junín OR "La Libertad" OR Lambayeque OR Lima OR Loreto OR "Madre de Dios" OR Moquegua OR Pasco OR Piura OR Puno OR "San Martín" OR Tacna OR Tumbes OR Ucayali ) OR SU ( Peru* OR Amazonas OR Ancash OR Apurímac OR Arequipa OR Ayacucho OR Cajamarca OR Cusco OR Callao OR Huancavelica OR Huánuco OR Ica OR Junín OR "La Libertad" OR Lambayeque OR Lima OR Loreto OR "Madre de Dios" OR Moquegua OR Pasco OR Piura OR Puno OR "San Martín" OR Tacna OR Tumbes OR Ucayali ) |
| #4 | S1 AND S2 AND S3 |

**RENATI**

The title of the undergraduate and postgraduate thesis contains the term burnout and its equivalents (in Spanish).

**SCIELO**

| #1 | burnout OR “emotional exhaustion” OR “professional exhaustion” OR “psychological exhaustion” |
| --- | --- |
| #2 | (burnout OR “emotional exhaustion” OR “professional exhaustion” OR “psychological exhaustion”) AND (peru* OR amazonas OR ancash OR apurímac OR arequipa OR ayacucho OR cajamarca OR cusco OR callao OR huancavelica OR huánuco OR ica OR junín OR “la libertad” OR lambayeque OR lima OR loreto OR “madre de dios” OR moquegua OR pasco OR piura OR puno OR “san martín” OR tacna OR tumbes OR ucayali) |
| #3 | (burnout OR “emotional exhaustion” OR “professional exhaustion” OR “psychological exhaustion”) AND (peru* OR amazonas OR ancash OR apurímac OR arequipa OR ayacucho OR cajamarca OR cusco OR callao OR huancavelica OR huánuco OR ica OR junín OR “la libertad” OR lambayeque OR lima OR loreto OR “madre de dios” OR moquegua OR pasco OR piura OR puno OR “san martín” OR tacna OR tumbes OR ucayali) AND (“Health Personnel” OR "Health Professional" OR "Health Care Providers" OR "Health Care Provider" OR "Healthcare Providers" OR "Healthcare Provider" OR "Healthcare Workers" OR "Healthcare Worker" OR "Pharmaceutical chemist" OR Nurs* OR Psychologist* OR Physician* OR Dentist* OR Nutritionist* OR Obstetrician* ) |
