# Supplementary material for: Prevalence and factors associated with burnout syndrome in Peruvian health professionals before the COVID-19 pandemic: A systematic review
Source: Heliyon. 2024 Apr 27;10(9):e30125. doi: 10.1016/j.heliyon.2024.e30125 (PMC11078627; doi:10.1016/j.heliyon.2024.e30125)
Supplement: Multimedia component 3 [file mmc3.docx]

Supplementary material 3. Data Extraction Sheet

| **General characteristics** | | | | | | | **Burnout diagnosis** | |
| --- | --- | --- | --- | --- | --- | --- | --- | --- |
| ID | First author | Link | Journal / University | Year of publication | Place (Regional) | Sample size | Measurement Burnout tool | Diagnostic criteria BDS (Yes or No) or ( slight, moderate or severe) |
|  |  |  |  |  |  |  |  |  |

| **Study Population** | | | | | | |
| --- | --- | --- | --- | --- | --- | --- |
| Population | Place (s) | Inclusion Criteria | Males (%) | Females (%) | Median age or mean (years) | Standard deviation (SD) |
|  |  |  |  |  |  |  |

| **Quantitative Burnout (Average)** | | | | | | | |
| --- | --- | --- | --- | --- | --- | --- | --- |
| Mean/Median total score | SD total score | Mean/Median of personal/ emotional  exhaustion (I) | SD of personal/  emotional exhaustion  (I) | Mean/Median of Despersonalization  (II) | SD of Despersonalization  (II) | Mean/Median of Personal  Fulfillment (III) | SD of Personal Fulfillment (III) |
|  |  |  |  |  |  |  |  |

| **Qualitative Burnout (%)** | | | | | | | | | | | | | | | |
| --- | --- | --- | --- | --- | --- | --- | --- | --- | --- | --- | --- | --- | --- | --- | --- |
| % of diagnosi s (Yes) | % of BSD  Sligh t | % of BSD  Moderat e | % of BSD  Sever e | % of personal/ emotional  exhaustion (AP) (Yes) | % AP  sligh t | % AP  Moderat e | % AP  Sever e | % de Despersonalizati on (D) (Yes) | % D  Sligh t | % D  Moderat e | % D  Sever e | % of  personal fulfillme nt (PF)  (Yes) | % RP  sligh t | % RP  Moderat e | % RP  Sever e |
|  |  |  |  |  |  |  |  |  |  |  |  |  |  |  |  |

| **Associated factors** | | | | **General characteristics** |
| --- | --- | --- | --- | --- |
| Associated factors with BSD significant only +  (Categories) | Associated factors with AP significant only +  (Categories) | Associated factors with D significant only +  (Categories) | Associated factors with PF significant only +  (Categories) | Study design (Transversal, CC, cohorte) |
|  |  |  |  |  |

| **Newcastle Ottawa** | | | | | | | |
| --- | --- | --- | --- | --- | --- | --- | --- |
| **Domain 1** | | | | **Domain 2** | **Domain 3** | | **Total** |
| Representative sample | Sample size justified | Not interviewed | Exposure measurement | Comparability | Outcome measurement | Statistical test |  |
|  |  |  |  |  |  |  |  |
